# Impact of community mental health center follow-up on psychiatric hospitalization burden in schizophrenia spectrum disorders: a retrospective mirror-image study

**DOI:** 10.1186/s12888-026-08285-6

**Published:** 2026-06-12

**Authors:** Haluk Usta, İzgi Ayçıl Gencan Yilmazer

**Affiliations:** 1 Department of Psychiatry, University of Health Sciences Erenköy Training and Research Hospital for Mental and Neurological Disorders, İstanbul, Türkiye; 2 Department of Psychiatry, University of Health Sciences Kartal Dr. Lütfi Kırdar City Hospital, İstanbul, Türkiye

**Keywords:** Schizophrenia, Community mental health center, Psychiatric rehabilitation, Hospitalization burden, Continuity of care, Mirror-image study, Severe mental illness, Community psychiatry

## Abstract

**Background:**

Community-based psychiatric rehabilitation services are increasingly used to reduce recurrent hospitalization and improve continuity of care among individuals with schizophrenia spectrum disorders. However, long-term real-world longitudinal data evaluating hospitalization outcomes following community mental health center follow-up remain limited. This study aimed to evaluate the impact of community mental health center (Toplum Ruh Sağlığı Merkezi [CMHC]) follow-up on psychiatric hospitalization burden among individuals with schizophrenia spectrum disorders using a retrospective mirror-image design.

**Methods:**

This retrospective longitudinal mirror-image study included 652 individuals with schizophrenia spectrum disorders followed at a community mental health center in Türkiye between 2015 and 2025. Annualized psychiatric hospitalization rates before and after CMHC enrollment were calculated for each participant. Hospitalization reduction scores were defined as the difference between pre-CMHC and post-CMHC annual hospitalization rates. Sociodemographic and clinical variables associated with hospitalization reduction were evaluated using nonparametric analyses, correlation analyses, and multivariable linear regression models.

**Results:**

Psychiatric hospitalization burden significantly decreased following CMHC enrollment. Mean annual hospitalization rates declined from 0.179 ± 0.204 admissions/year before CMHC follow-up to 0.061 ± 0.159 admissions/year after enrollment (*p* < 0.001). The mean hospitalization reduction score was 0.118 ± 0.218, corresponding to a large effect size. In multivariable regression analyses, higher pre-CMHC annual hospitalization rates independently predicted higher post-CMHC hospitalization rates (β = 0.315, *p* < 0.001), whereas longer CMHC follow-up duration was independently associated with lower post-CMHC hospitalization rates (β = −0.119, *p* = 0.004). Hospitalization reduction scores were positively correlated with both CMHC follow-up duration (*r* = 0.308, *p* < 0.001) and total number of psychiatric hospitalizations (*r* = 0.386, *p* < 0.001). Most sociodemographic and clinical variables were not significantly associated with differential hospitalization reduction outcomes.

**Conclusions:**

Long-term follow-up within a community mental health center was associated with significant reductions in psychiatric hospitalization burden among individuals with schizophrenia spectrum disorders. Sustained rehabilitation follow-up and continuity of care may contribute to improved long-term clinical stabilization and reduced inpatient psychiatric service utilization. These findings suggest that sustained CMHC follow-up may be associated with reduced psychiatric hospitalization burden in routine clinical practice.

**Clinical Trial Registration:**

Non applicable.

## Introduction

Schizophrenia spectrum disorders are severe and chronic psychiatric illnesses characterized by recurrent psychotic episodes, impaired social and occupational functioning, and substantial long-term disability. Despite advances in pharmacological and psychosocial treatment strategies, many patients continue to experience recurrent relapses and repeated psychiatric hospitalizations throughout the course of illness. Psychiatric rehospitalization is associated with worsening functional outcomes, reduced quality of life, increased caregiver burden, premature mortality, and markedly elevated healthcare expenditures [1–3]. Accordingly, hospitalization burden has emerged as one of the most important indicators of disease severity, treatment continuity, and long-term prognosis in schizophrenia spectrum disorders [1, 2].

Over recent decades, the transition from institutionalized psychiatric care toward community-based mental health systems has substantially transformed the management of severe mental illnesses. Contemporary recovery-oriented mental health policies increasingly emphasize continuity of care, psychosocial rehabilitation, community integration, multidisciplinary follow-up, and individualized long-term support models [4, 5]. Within this framework, Community Mental Health Centers (CMHCs; Toplum Ruh Sağlığı Merkezleri [TRSM] in Türkiye) provide comprehensive multidisciplinary rehabilitation services including psychiatric follow-up, medication monitoring, psychoeducation, psychosocial interventions, family counseling, crisis management, and social reintegration programs. The primary goals of these services are to improve long-term clinical stability, reduce relapse frequency, and minimize recurrent psychiatric hospitalization burden [5, 6].

Previous studies have demonstrated that community-based psychiatric rehabilitation services may reduce inpatient psychiatric service utilization and improve psychosocial outcomes among individuals with severe mental illness. Dalton-Locke et al., in a systematic review of rehabilitation psychiatry services, reported that structured rehabilitation-oriented mental health interventions were associated with reductions in long-term inpatient psychiatric care and improvements in community functioning [7]. Similarly, Killaspy and Dalton-Locke emphasized that sustained longitudinal rehabilitation services and continuity of care represent critical components of successful recovery-oriented mental healthcare systems [5]. McPherson et al. further demonstrated that supported accommodation and community rehabilitation interventions may contribute to improved psychosocial outcomes and reduced reliance on inpatient psychiatric treatment among individuals with severe psychotic disorders [8]. In addition, recent evidence suggests that long-term continuity of care and coordinated multidisciplinary follow-up may play important roles in reducing healthcare utilization and improving functional recovery in schizophrenia spectrum disorders [9–11].

Despite growing evidence supporting community-based psychiatric rehabilitation, the literature remains heterogeneous regarding study design, follow-up duration, outcome measures, and rehabilitation service structures [7–9]. In particular, long-term real-world longitudinal studies evaluating psychiatric hospitalization burden before and after enrollment in community mental health services remain limited. Most available studies have focused on short-term outcomes, supported housing models, or specific rehabilitation interventions rather than longitudinal changes in hospitalization burden within the same individuals. Furthermore, evidence from middle-income countries and national community psychiatry systems outside Western Europe and North America remains relatively scarce [7–12].

In Türkiye, CMHCs have become an increasingly important component of psychiatric rehabilitation following nationwide implementation of community-based mental health policies. Previous Turkish studies have primarily focused on treatment adherence, psychosocial functioning, and patient satisfaction among CMHC users [13, 14]. However, long-term longitudinal studies specifically evaluating psychiatric hospitalization burden among individuals with schizophrenia spectrum disorders remain limited. Moreover, retrospective mirror-image studies comparing hospitalization patterns before and after CMHC follow-up within the same individuals are exceedingly rare.

Therefore, the present study aimed to evaluate the longitudinal impact of CMHC follow-up on psychiatric hospitalization burden among individuals with schizophrenia spectrum disorders using a retrospective mirror-image design. In addition, we sought to identify clinical factors associated with greater reductions in hospitalization burden following long-term community mental health follow-up.

## Methods

### Study design and setting

This retrospective longitudinal mirror-image study was conducted at the Kadıköy CMHC affiliated with Erenköy Mental and Neurological Diseases Training and Research Hospital. The study aimed to evaluate the longitudinal impact of community-based psychiatric rehabilitation services on psychiatric hospitalization burden among individuals diagnosed with schizophrenia spectrum disorders.

The study was designed using a mirror-image model. In this design, the duration between the patient’s enrollment in the CMHC and the most recent follow-up date was first calculated, and all psychiatric hospitalizations occurring during this follow-up period were recorded. Subsequently, an equivalent time interval of identical duration was retrospectively determined prior to CMHC enrollment by counting backward from the enrollment date. In this way, the pre-CMHC and post-CMHC periods were standardized in terms of duration, thereby creating a symmetrical “mirror-image” comparison model.

For example, if a patient diagnosed with schizophrenia in 2000 was enrolled in the CMHC in 2019, the duration between 2019 and the most recent follow-up date was calculated. An equivalent retrospective period of the same duration (for example, 2013–2019) was then identified before CMHC enrollment. These two periods were considered symmetrical counterparts within the mirror-image framework, and psychiatric hospitalization frequencies during both periods were systematically compared and recorded.

The mirror-image design compared psychiatric hospitalization outcomes during the pre-CMHC period and the post-CMHC follow-up period within the same individuals, thereby minimizing interindividual variability and allowing each participant to serve as their own control. This approach has been widely used in psychiatric service research to evaluate real-world outcomes associated with longitudinal community mental health interventions.

The study protocol was approved by the Scientific Research Ethics Committee of University of Health Sciences Erenköy Mental and Neurological Diseases Training and Research Hospital prior to data collection (Decision No: 102; Meeting No: 2025/22; Approval Date: 27 November 2025). Because of the retrospective nature of the study and the use of anonymized medical records, the requirement for informed consent was waived.

### Participants

The study included adult patients with schizophrenia spectrum disorders who were registered and followed at the Kadıköy CMHC between September 2015 and December 2025. Diagnoses were established by treating psychiatrists according to DSM-5-TR criteria and confirmed through electronic medical records.

All participants were longitudinally followed by the same attending psychiatrist (HU) from the time of CMHC enrollment onward, although members of the multidisciplinary treatment team could vary over time.

### Inclusion criteria

Patients were included if they:

1. were aged ≥ 18 years,
2. had a diagnosis of schizophrenia spectrum disorder,
3. had available hospitalization records for both the pre-CMHC and post-CMHC periods,
4. had accessible longitudinal follow-up records within the CMHC system.

### Exclusion criteria

Patients were excluded if they:

1. had incomplete hospitalization records,
2. had less than one year of CMHC follow-up.
3. had severe neurological or neurodegenerative disorders that could substantially affect psychiatric hospitalization patterns.

Incomplete hospitalization records were defined as missing electronic medical record data required for the calculation of hospitalization outcomes or key study variables.

The Kadıköy CMHC was established in 2015 and, since its implementation, a total of 1,699 patients have been registered within the system. Among these, 1,007 patients were actively receiving CMHC follow-up at the time of data collection. All patient records were retrospectively reviewed, and according to the predefined inclusion and exclusion criteria, a total of 652 patients with complete and analyzable data were included in the final analyses.

### Community mental health center (CMHC) services

The Kadıköy CMHC provides multidisciplinary, community-based psychiatric rehabilitation services for individuals with severe mental illness. Services include regular psychiatric assessment, pharmacological treatment monitoring, psychoeducation, psychosocial rehabilitation, family counseling, crisis intervention, social support interventions, and community reintegration programs.

Pharmacological monitoring included administration of long-acting injectable antipsychotics and laboratory monitoring of medications such as clozapine and lithium. Psychoeducation was provided to both patients and family members. Rehabilitation activities focused on social skills, community participation, and occupational functioning.

The multidisciplinary team consisted of one attending psychiatrist, five psychiatric nurses, one psychologist, one occupational therapist, one social worker, and rotating psychiatry residents. Additional rehabilitation activities included vocational, artistic, and social skills programs delivered by external instructors.

Patients were followed longitudinally through scheduled outpatient visits and individualized rehabilitation planning. Continuity of care was maintained through regular clinical monitoring and coordinated multidisciplinary interventions.

### Data Collection

Data were retrospectively extracted from electronic medical records and CMHC registry files. The following variables were collected:

- demographic characteristics (age, sex, marital status, education, employment status, living conditions),
- clinical variables (age at illness onset, illness duration, family history of psychosis, suicide attempt history, substance use history),
- psychosocial variables (social support and socioeconomic status),
- CMHC follow-up duration,
- psychiatric hospitalization history.

Hospitalization-related variables included:

- total number of psychiatric hospitalizations,
- annualized hospitalization rate before CMHC enrollment,
- annualized hospitalization rate after CMHC enrollment,
- hospitalization reduction score.

Perceived social support and socioeconomic status were retrospectively categorized based on routine clinical assessments documented in CMHC records. Social support ratings were determined according to living arrangements, family involvement, and attendance at follow-up visits. Socioeconomic status was assessed using information regarding household income, housing status, household size, and residential characteristics and categorized into five levels (very poor, poor, moderate, good, and very good).

### Definition of hospitalization outcomes

To account for variability in follow-up duration, annualized hospitalization rates were calculated for both periods.

Annualized hospitalization rates were preferred over raw admission counts to account for interindividual variability in follow-up duration and to provide standardized longitudinal comparisons between pre-CMHC and post-CMHC periods.

### Pre-CMHC annual hospitalization rate

The pre-CMHC hospitalization rate was calculated by dividing the number of psychiatric hospitalizations occurring during the individualized mirror pre-CMHC period by the duration of that period in years. For each participant, the pre-CMHC observation window was defined as a period identical in duration to the post-CMHC follow-up period, thereby preserving the symmetry of the mirror-image design.

### Post-CMHC annual hospitalization rate

The post-CMHC hospitalization rate was calculated by dividing the number of psychiatric hospitalizations during CMHC follow-up by the individual follow-up duration in years.

### Hospitalization reduction score

The primary outcome measure was the hospitalization reduction score, calculated as:

$$\eqalign{ & Hospitalization\,Reduction\,Score\, = \cr & Pre\, - \,CMHC{\rm{ }}\,Annual\,Hospitalization\,Rate\, - \cr & Post\, - \,CMHC\,Annual\,Hospitalization\,Rate \cr}$$

Positive values indicated a reduction in psychiatric hospitalization burden following CMHC enrollment.

### Statistical analysis

All statistical analyses were performed using IBM SPSS Statistics version 31.0 (IBM Corp., Armonk, NY, USA).

Continuous variables were evaluated for normality using the Kolmogorov–Smirnov and Shapiro–Wilk tests, as well as histogram and Q-Q plot inspections. Since most continuous variables demonstrated non-normal distributions, nonparametric statistical methods were preferred when appropriate.

Continuous variables were presented as mean ± standard deviation (SD) or median and interquartile range (IQR), depending on distribution characteristics. Categorical variables were expressed as frequencies and percentages.

The primary comparison between pre-CMHC and post-CMHC annual hospitalization rates was performed using the Wilcoxon signed-rank test. Effect size for paired comparisons was calculated using the standardized test statistic (r = Z/√N).

Between-group comparisons for hospitalization reduction scores were performed using the Mann–Whitney U test.

Correlation analyses between hospitalization reduction scores and continuous clinical variables were evaluated using Spearman rank correlation coefficients.

To identify independent predictors of hospitalization reduction, multivariable linear regression analysis was performed. Variables with clinical relevance and/or statistical significance in univariate analyses were included in the multivariable model. Multicollinearity was assessed using variance inflation factor (VIF) and tolerance statistics.

A two-sided p-value < 0.05 was considered statistically significant throughout all analyses.

## Results

### Patient characteristics

A total of 652 individuals with schizophrenia spectrum disorders were included in the final analyses. The mean age of the study population was 47.5 ± 11.8 years, and 57.2% of the participants were male. The median age at illness onset was 23 years (IQR: 11), while the median duration of illness was 264 months (IQR: 192). The mean lifetime number of psychiatric hospitalizations was 3.83 ± 4.15.

Regarding sociodemographic characteristics, 67.9% of the patients were single, and 51.4% were unemployed. Most participants lived with family members (88.5%) and reported moderate socioeconomic status (65.3%). A history of suicide attempt was present in 38.0% of the cohort, while substance use history was identified in 11.3% of patients. Detailed demographic and clinical characteristics are presented in Table 1.

**Table 1:** Baseline demographic and clinical characteristics of the study population (*N* = 652)

Variable	Value
**Age**,** years**	47.5 ± 11.8
Median age (IQR)	48 (17)
Age range	20–93
**Age at illness onset**,** years**	25.9 ± 9.1
Median age at onset (IQR)	23 (11)
**Total illness duration**,** months**	269.9 ± 128.8
Median illness duration (IQR)	264 (192)
**Total lifetime psychiatric hospitalizations**	3.83 ± 4.15
Median hospitalization count (IQR)	2 (4)
**Sex**	
Female	279 (42.8%)
Male	373 (57.2%)
**Marital status**	
Single	443 (67.9%)
Married	94 (14.4%)
Widowed/divorced	115 (17.6%)
**Employment status**	
Unemployed	335 (51.4%)
Employed	225 (34.5%)
Retired	43 (6.6%)
Disability retirement	49 (7.5%)
**Education level**	
No formal education	15 (2.3%)
Primary school	250 (38.3%)
Secondary education	220 (33.7%)
University	167 (25.6%)
**Active smokers**	390 (59.8%)
**Alcohol use**,** active or in the past**	94 (14.4%)
**Substance use history**	74 (11.3%)
**Suicide attempt history**	248 (38.0%)
**Family history of psychosis**	363 (55.7%)
**Living conditions**	
Living with family	577 (88.5%)
Living alone	67 (10.3%)
Nursing home/other	8 (1.2%)
**Regular CMHC attendance**	629 (96.5%)
**Perceived social support**	
Very poor	19 (2.9%)
Poor	190 (29.1%)
Moderate	293 (44.9%)
Good	143 (21.9%)
Very good	7 (1.1%)
**Socioeconomic status**	
Very poor	7 (1.1%)
Poor	85 (13.0%)
Moderate	426 (65.3%)
Good	126 (19.3%)
Very good	8 (1.2%)

Data are presented as mean ± standard deviation, median (interquartile range), or n (%), as appropriate

### Changes in psychiatric hospitalization burden

Psychiatric hospitalization burden significantly decreased following CMHC enrollment (Table 2). The median annualized psychiatric hospitalization rate decreased from 0.10 admissions/year (IQR: 0.10) during the pre-CMHC period to 0.00 admissions/year (IQR: 0.00) during the post-CMHC follow-up period.

**Table 2:** Changes in psychiatric hospitalization burden before and after CMHC follow-up

Variable	Mean ± SD	Median (IQR)	Min–Max	*p*-value
Pre-CMHC annual hospitalization rate	0.179 ± 0.204	0.10 (0.10)	0.00–1.50	< 0.001*
Post-CMHC annual hospitalization rate	0.061 ± 0.159	0.00 (0.00)	0.00–1.20	
Hospitalization reduction score	0.118 ± 0.218	0.10 (0.20)	-1.20–1.18	

*Wilcoxon signed-rank test: Z = -15.054, *p* < 0.001; effect size *r* = 0.59. Data are presented as mean ± standard deviation, median (interquartile range), and minimum–maximum values

Similarly, the mean annual hospitalization rate decreased from 0.179 ± 0.204 before CMHC enrollment to 0.061 ± 0.159 after CMHC follow-up. Wilcoxon signed-rank analysis demonstrated a statistically significant reduction in hospitalization burden following CMHC enrollment (Z = -15.054, *p* < 0.001), corresponding to a large effect size (*r* = 0.59).

Overall, hospitalization reduction scores were positive in 91.4% of the cohort (596/652), indicating reduced psychiatric hospitalization burden following CMHC enrollment, whereas 8.6% of patients demonstrated negative scores. Comparisons between the positive and negative hospitalization reduction groups revealed that poorer social support, history of suicide attempts, active smoking status, lower age at illness onset, and higher total number of psychiatric hospitalizations were significantly more common in the negative-score group.

**Fig. 1:**
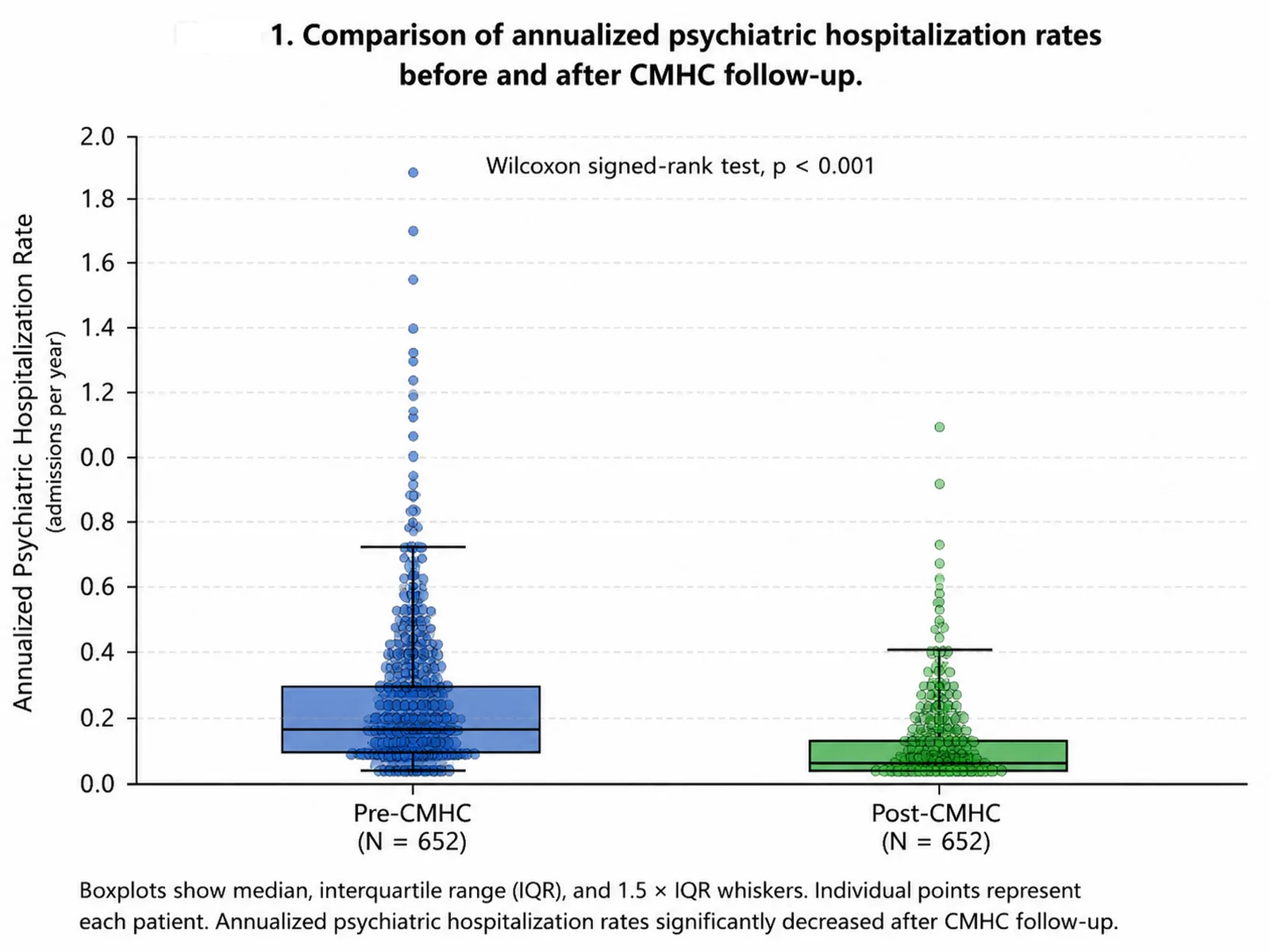
Comparison of annualized psychiatric hospitalization rates before and after CMHC follow-up. Annualized psychiatric hospitalization rates significantly decreased following enrollment in community-based psychiatric rehabilitation services (Wilcoxon signed-rank test, *p* < 0.001)

The distributional changes in annualized psychiatric hospitalization rates before and after CMHC follow-up are illustrated in Fig. 1.

The mean hospitalization reduction score was 0.118 ± 0.218, with a median reduction of 0.10 (IQR: 0.20), indicating a substantial overall decrease in annual psychiatric hospitalization burden after initiation of community-based psychiatric rehabilitation services.

### Factors associated with hospitalization reduction

Between-group analyses demonstrated that hospitalization reduction scores did not significantly differ according to sex (*p* = 0.468), smoking status (*p* = 0.587), alcohol use history (*p* = 0.534), suicide attempt history (*p* = 0.415), family history of psychosis (*p* = 0.463), or regular CMHC attendance status (*p* = 0.112).

However, patients with a history of substance use demonstrated significantly greater hospitalization reduction scores compared with those without substance use history (mean reduction: 0.162 ± 0.301 vs. 0.113 ± 0.205, respectively; Mann–Whitney U test, *p* = 0.028).

Detailed subgroup analyses are summarized in Table 3.

**Table 3:** Factors associated with hospitalization reduction following CMHC follow-up

Variable	Group	Median (IQR)	Mean ± SD	*p*-value
Sex	Female	0.10 (0.00–0.20)	0.12 ± 0.23	0.468
	Male	0.10 (0.00–0.20)	0.12 ± 0.21	
Smoking history	No	0.10 (0.00–0.20)	0.12 ± 0.16	0.587
	Yes	0.10 (0.00–0.20)	0.12 ± 0.25	
Alcohol use history	No	0.10 (0.00–0.20)	0.12 ± 0.21	0.534
	Yes	0.10 (0.01–0.20)	0.13 ± 0.25	
Substance use history	No	0.10 (0.00–0.20)	0.11 ± 0.20	0.028
	Yes	0.10 (0.09–0.27)	0.16 ± 0.30	
Suicide attempt history	No	0.10 (0.00–0.20)	0.11 ± 0.19	0.415
	Yes	0.10 (0.00–0.20)	0.13 ± 0.26	
Family history of psychosis	No	0.10 (0.00–0.20)	0.12 ± 0.22	0.463
	Yes	0.10 (0.00–0.20)	0.12 ± 0.21	
Regular CMHC attendance	No	0.10 (0.00–0.10)	0.08 ± 0.33	0.112
	Yes	0.10 (0.00–0.20)	0.12 ± 0.21	

*p*-values were calculated using the Mann–Whitney U test. Data are presented as median (interquartile range) and mean ± standard deviation

### Correlation analyses

Spearman rank correlation analyses demonstrated that hospitalization reduction scores were positively correlated with CMHC follow-up duration (*r* = 0.308, *p* < 0.001) and total number of psychiatric hospitalizations (*r* = 0.386, *p* < 0.001) (Table 4).

**Table 4:** Correlations between hospitalization reduction scores and clinical variables

Variable	Hospitalization Reduction Scores	
	Correlation coefficient (*r*)	*p*-value
Age	0.022	0.575
Age at illness onset	-0.038	0.329
Total illness duration	0.06	0.128
CMHC follow-up duration	0.308	< 0.001
Total psychiatric hospitalization count	0.386	< 0.001

Correlation analyses were performed using Spearman rank correlation coefficients

No significant correlations were identified between hospitalization reduction scores and age (*p* = 0.575), age at illness onset (*p* = 0.329), or total illness duration (*p* = 0.128).

**Fig. 2:**
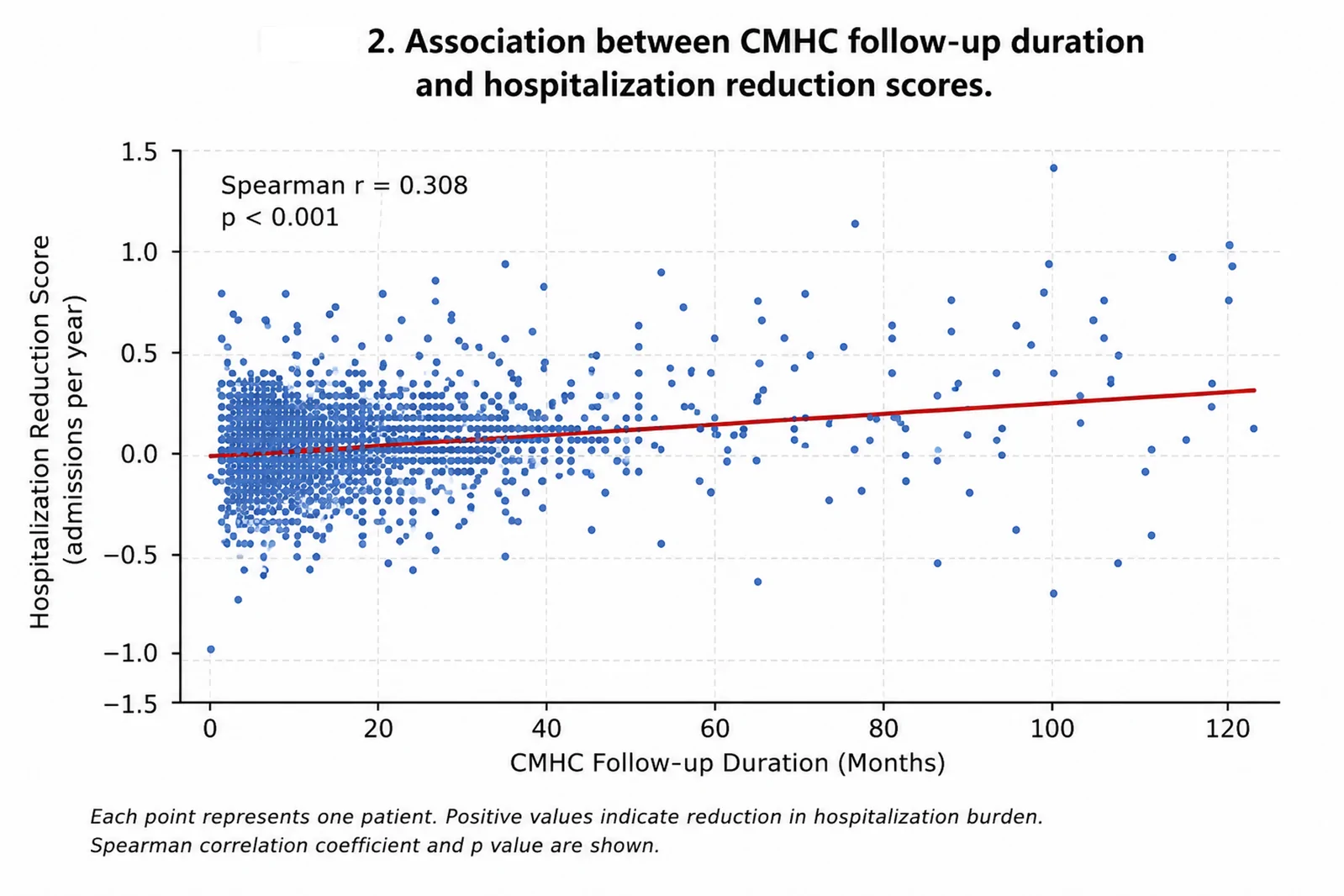
Association between CMHC follow-up duration and hospitalization reduction scores. Longer duration of community-based psychiatric rehabilitation follow-up was significantly associated with greater reductions in psychiatric hospitalization burden (Spearman *r* = 0.308, *p* < 0.001)

As illustrated in Fig. 2, longer engagement with community-based psychiatric rehabilitation services was associated with greater reductions in psychiatric hospitalization burden.

### Multivariable predictors of post-CMHC hospitalization rate

In response to concerns regarding regression-to-the-mean and mathematical coupling, an additional multivariable linear regression analysis was performed using post-CMHC annual hospitalization rate as the dependent variable and pre-CMHC annual hospitalization rate as a covariate.

The model was statistically significant overall (F = 13.48, *p* < 0.001) and explained 10.3% of the variance in post-CMHC hospitalization rates (adjusted R² = 0.103).

Higher pre-CMHC annual hospitalization rates independently predicted higher post-CMHC hospitalization rates (β = 0.315, *p* < 0.001). Importantly, longer CMHC follow-up duration remained independently associated with lower post-CMHC hospitalization rates after adjustment for baseline hospitalization burden (β = -0.119, *p* = 0.004).

Age, illness duration, substance use history, and sex were not independently associated with post-CMHC hospitalization rates. Detailed results of the multivariable regression analysis are presented in Table 5.

**Table 5:** Multivariable linear regression analysis predicting post-CMHC annual hospitalization rate

Variable	B	Standard Error	Standardized β	95% CI	*p*-value
Constant	0.072	0.036	–	0.002 to 0.142	0.044
Pre-CMHC annual hospitalization rate	0.246	0.031	0.315	0.184 to 0.308	< 0.001
CMHC follow-up duration (months)	-0.001	< 0.001	-0.119	-0.002 to -0.001	0.004
Age (years)	-0.001	0.001	-0.085	-0.002 to < 0.001	0.098
Total illness duration (months)	< 0.001	< 0.001	0.079	< 0.001 to < 0.001	0.122
Substance use history	0.032	0.02	0.063	-0.007 to 0.071	0.110
Sex (male vs. female)	0.005	0.012	0.014	-0.018 to 0.029	0.714

**Model statistics**: Adjusted R² = 0.103, F = 13.482, *p* < 0.001. **Dependent variable**: Post-CMHC annual hospitalization rate. **Abbreviations**: B, unstandardized regression coefficient; β, standardized regression coefficient; CI, confidence interval; CMHC, Community Mental Health Center.

## Discussion

In this retrospective longitudinal mirror-image study, we demonstrated that long-term follow-up within a CMHC was associated with a substantial reduction in psychiatric hospitalization burden among individuals with schizophrenia spectrum disorders. Both annualized hospitalization rates and hospitalization reduction scores showed significant improvement following CMHC enrollment, with a large observed effect size. In addition, longer CMHC follow-up duration and greater baseline hospitalization burden independently predicted larger reductions in psychiatric hospitalization rates. These findings suggest that sustained CMHC follow-up may be associated with lower psychiatric hospitalization burden in routine clinical practice. Our findings are consistent with the growing body of literature emphasizing the importance of community-based psychiatric rehabilitation and continuity of care in schizophrenia spectrum disorders. Contemporary recovery-oriented mental health systems increasingly prioritize multidisciplinary care models aimed at reducing recurrent hospitalization, improving psychosocial functioning, and promoting long-term community integration [4, 5]. Previous studies have shown that rehabilitation-oriented mental health services, supported accommodation programs, and coordinated community follow-up may reduce hospitalization rates and improve functional outcomes among individuals with severe psychotic disorders [7–9]. In particular, Dalton-Locke et al. demonstrated that structured rehabilitation services were associated with reductions in long-term inpatient psychiatric service utilization among patients with complex psychosis [7]. Similarly, Killaspy and Dalton-Locke emphasized that continuity of rehabilitation care and sustained therapeutic engagement are central components of effective recovery-oriented mental healthcare systems [5]. The present study extends these findings by providing long-term real-world mirror-image data from a large cohort within a national community psychiatry system.

These findings may also be interpreted within the broader context of international community psychiatry models. For example, the Italian community mental health system is widely regarded as one of the most established community-based care models worldwide, emphasizing continuity of care, multidisciplinary rehabilitation, and social inclusion. Nevertheless, recent evaluations have highlighted ongoing challenges related to resource allocation, workforce capacity, and regional variability in service delivery [15]. The present findings provide additional support for the potential value of sustained community-based follow-up in reducing hospitalization burden across diverse healthcare settings.

One of the most clinically important findings of this study was the marked reduction in annualized psychiatric hospitalization rates following CMHC enrollment. Psychiatric rehospitalization remains a major challenge in schizophrenia spectrum disorders because recurrent admissions are closely associated with illness instability, functional deterioration, impaired quality of life, caregiver burden, and increased healthcare expenditures [1–3]. Reducing hospitalization burden therefore represents not only a marker of improved clinical stability, but also an important indicator of healthcare system effectiveness and rehabilitation success. The substantial decline in hospitalization rates observed in our cohort suggests that structured longitudinal community follow-up may contribute to stabilization of chronic psychotic illness trajectories in routine clinical practice.

Another notable finding was the positive association between CMHC follow-up duration and hospitalization reduction scores. As illustrated in Fig. 2, longer engagement with community-based psychiatric rehabilitation services was associated with progressively greater reductions in psychiatric hospitalization burden. This finding supports the concept that continuity of care and sustained multidisciplinary follow-up are critical determinants of successful psychiatric rehabilitation outcomes. Recovery-oriented rehabilitation models emphasize long-term therapeutic relationships, individualized rehabilitation planning, psychosocial support, regular monitoring, and proactive relapse prevention strategies rather than episodic crisis-oriented interventions alone [5, 8]. Our findings therefore suggest that the benefits of CMHC-based rehabilitation may accumulate over time through sustained longitudinal stabilization and therapeutic continuity.

Although patients with higher baseline hospitalization burden showed greater reductions in hospitalization rates, this finding should be interpreted cautiously because regression-to-the-mean and mathematical coupling may partially contribute to this association. Similar observations have been reported in studies of psychiatric readmission, where prior hospitalization burden consistently emerges as one of the strongest predictors of future service utilization [7, 8]. In a recent study of patients with psychotic disorders, Besana et al. identified previous psychiatric admissions as a major predictor of subsequent readmission risk, highlighting the persistent influence of baseline service utilization on future hospitalization trajectories [16]. From a public mental health perspective, this finding is especially important because patients with recurrent admissions constitute a disproportionately high burden on psychiatric healthcare systems and often require extensive long-term healthcare resource [8, 12–15].

Although patients with higher baseline hospitalization burden showed greater reductions in hospitalization rates, this finding should be interpreted cautiously because regression-to-the-mean and mathematical coupling may partially contribute to this association. Similar observations have been reported in studies of psychiatric readmission, where prior hospitalization burden consistently emerges as one of the strongest predictors of future service utilization [7, 8]. In a recent study of patients with psychotic disorders, Besana et al. similarly identified previous psychiatric admissions as a major predictor of subsequent readmission risk among individuals with psychotic disorders [16]. To address this concern, we performed an additional multivariable regression analysis using post-CMHC annual hospitalization rate as the outcome and baseline hospitalization rate as a covariate. In this model, longer CMHC follow-up duration remained independently associated with lower post-CMHC hospitalization rates, even after adjustment for baseline hospitalization burden. This finding suggests that the association between sustained CMHC engagement and reduced hospitalization burden cannot be fully explained by baseline severity alone. Nevertheless, regression-to-the-mean remains an inherent limitation of mirror-image study designs and should be considered when interpreting the observed associations [17].

Subgroup analyses demonstrated that hospitalization reduction scores were generally consistent across most sociodemographic and clinical subgroups. Sex, smoking status, alcohol use history, suicide attempt history, family history of psychosis, and regular CMHC attendance status were not significantly associated with differential hospitalization reduction outcomes. However, patients with substance use history demonstrated greater hospitalization reduction scores in univariate analyses. Although this association did not remain significant after multivariable adjustment, it may suggest that patients with greater baseline psychosocial instability have more potential for measurable improvement following structured rehabilitation interventions. Nevertheless, this finding should be interpreted cautiously because of the retrospective observational design and the relatively smaller size of the substance-use subgroup.

The present study has several important strengths. First, the large sample size and long follow-up duration provide robust real-world longitudinal data regarding psychiatric rehabilitation outcomes in schizophrenia spectrum disorders. Second, the mirror-image design minimized interindividual variability by allowing each participant to serve as their own control. Third, the use of annualized hospitalization rates enabled standardized comparisons despite variability in follow-up duration. Fourth, the integration of longitudinal hospitalization data with community rehabilitation follow-up variables allowed evaluation of clinically meaningful real-world outcomes. Additionally, only 652 of 1,699 registered patients met the eligibility criteria and had complete analyzable data. Therefore, selection bias cannot be excluded, as individuals who disengaged from services or had incomplete records may differ systematically from those included in the analyses. Furthermore, part of the study period overlapped with the COVID-19 pandemic, during which psychiatric admission practices and healthcare utilization patterns may have changed. Finally, the study contributes valuable evidence from a middle-income country community psychiatry system, addressing an important gap in the international rehabilitation psychiatry literature.

Several limitations should also be acknowledged. First, the retrospective single-center design limits causal inference and may reduce generalizability to other healthcare settings. Because longitudinal pharmacological treatment changes could not be standardized retrospectively, part of the observed improvement may also reflect optimization of antipsychotic treatment during follow-up. Second, symptom severity scales, cognitive assessments, medication adherence measures, and detailed psychosocial functioning data were not consistently available in retrospective records. Third, changes in pharmacological treatment strategies during long-term follow-up could not be fully controlled. In addition, unmeasured confounding variables related to family support, treatment adherence, social functioning, and healthcare accessibility may have influenced hospitalization outcomes. Furthermore, because hospitalization burden was used as the primary outcome measure, the study could not directly evaluate broader recovery domains such as occupational functioning, social participation, or quality of life. In addition, mirror-image designs may partially be influenced by regression-to-the-mean effects, whereby patients with initially high hospitalization burden may naturally demonstrate some reduction over time independent of intervention effects. Therefore, prospective multicenter longitudinal studies incorporating standardized functional and symptom-based assessments are needed to further clarify the long-term clinical, psychosocial, and economic effectiveness of community-based psychiatric rehabilitation services.

In conclusion, long-term follow-up within a community mental health center was associated with substantial reductions in psychiatric hospitalization burden among individuals with schizophrenia spectrum disorders. Longer duration of rehabilitation follow-up and greater baseline hospitalization burden independently predicted greater improvement in hospitalization outcomes. These findings suggest that CMHC follow-up may be associated with reduced psychiatric hospitalization burden in routine clinical practice and highlight the importance of continuity of care in severe mental illness management. Further prospective multicenter studies are warranted to evaluate the long-term clinical, functional, and economic impact of CMHC-based psychiatric rehabilitation models.

## Data Availability

The datasets generated and/or analyzed during the current study are not publicly available because they contain sensitive patient information, but anonymized data may be available from the corresponding author on reasonable request and with institutional permission.

## Abbreviations

CMHCs: Community Mental Health Centers
DSM-5-TR: Diagnostic and Statistical Manual of Mental Disorders, Fifth Edition, Text Revision
NY: New York
USA: United States of America
SPSS: Statistical Package for the Social Sciences
VIF: Variance Inflation Factor
IQR: Interquartile Range
B: Unstandardized regression coefficient
β: Standardized regression coefficient
CI: Confidence interval
